# The Involvement of Natural Polyphenols in Molecular Mechanisms Inducing Apoptosis in Tumor Cells: A Promising Adjuvant in Cancer Therapy

**DOI:** 10.3390/ijms24021680

**Published:** 2023-01-14

**Authors:** Adele Chimento, Arianna De Luca, Maria D’Amico, Francesca De Amicis, Vincenzo Pezzi

**Affiliations:** 1 Department of Pharmacy, Health and Nutritional Sciences, University of Calabria, 87036 Rende, CS, Italy; 2 Health Center, University of Calabria, 87036 Rende, CS, Italy

**Keywords:** cancer prevention, apoptosis, polyphenols, flavonoids, non-flavonoids

## Abstract

Various literature data show how a diet rich in vegetables could reduce the incidence of several cancers due to the contribution of the natural polyphenols contained in them. Polyphenols are attributed multiple pharmacological actions such as anti-inflammatory, anti-oxidant, antibiotic, antiseptic, anti-allergic, cardioprotective and even anti-tumor properties. The multiple mechanisms involved in their anti-tumor action include signaling pathways modulation associated with cell proliferation, differentiation, migration, angiogenesis, metastasis and cell death. Since the dysregulation of death processes is involved in cancer etiopathology, the natural compounds able to kill cancer cells could be used as new anticancer agents. Apoptosis, a programmed form of cell death, is the most potent defense against cancer and the main mechanism used by both chemotherapy agents and polyphenols. The aim of this review is to provide an update of literature data on the apoptotic molecular mechanisms induced by some representative polyphenol family members in cancer cells. This aspect is particularly important because it may be useful in the design of new therapeutic strategies against cancer involving the polyphenols as adjuvants.

## 1. Introduction

Cancer is a complex pathological process mainly characterized by altered cell death and the uncontrolled proliferation of cells, which, through blood or lymphatic diffusion, can also give rise to distant metastases [1]. The continuing growth of global cancer incidence is rapidly outpacing the ability to control and block this disease [2]. In 2020, over 19 million new cases were diagnosed, and more than 10 million deaths were recorded [3]. Most cancers can have multiple causes of which the trigger is difficult to identify; generally, environmental [4], infectious [5] and hereditary factors [6] play a key role in cancer onset [7].

Cancer hallmarks include a set of functional capabilities acquired by human cells that allow the transition from a normal physiological state to a malignant neoplastic one [1]. The sustained proliferative signaling, evasion of growth suppressors and immune destruction, replicative immortality, angiogenesis, invasion, metastasis, as well as cell death resistance and reprogramming cellular metabolism, represent the main cancer hallmarks [1]. In particular, the loss of balance between cell proliferation and cell death can lead to cancer onset [8]. When death signals are missing, the uncontrolled cell proliferation occurs leading to different cancer types [9]. The main mechanism for cell death is represented by apoptosis, a complex process genetically controlled and evolutionarily conserved; it primarily consists of two main pathways, the extrinsic death-receptor (DR)-mediated one and the mitochondria-mediated one, both of which could lead to the same terminal execution pathway [10]. In addition, the inflammation, genome instability and mutations, as well as non-mutational epigenetic reprogramming and polymorphic variations in organ/tissue microbiomes, are involved in activating the above functional hallmark capabilities necessary for tumor growth and progression [1]. Genomic instability, resulting from increased mutation rate and DNA sequence alterations, is a major enabling cancer mechanism [11,12]. There is a close relationship between genomic integrity and cell death regulation [13,14]; genomic instability can lead to mutations or changes in the expression levels of cell death main regulators, while dysregulated apoptosis can promote genomic instability [13]. In fact, the telomere dysfunction [15], DNA double strand breaks [16], polyploidy [17] or abnormal mitoses [18] can directly trigger apoptosis through specific pathways; on the other hand, the inhibited apoptosis increases chromosomal instability risk and directs cells to survive for a long time. Therefore, apoptosis can prevent genetically damaged cells from surviving and thus can protect against genomic instability by limiting the viable mutants’ number. In this regard, the knowledge of dysregulation apoptosis and the signaling cascades triggering apoptosis in response to anticancer agents provides information on new effective therapeutic approaches based on molecular-targeted anticancer therapies.

Cancer therapy, indeed, has the main goal of blocking cancer cell survival and inducing death in all clonogenic cells. Current therapies such as chemotherapy [19] and radiotherapy [20] are capable of inducing cell death through various molecular mechanisms; however, they have disadvantages, including chemo- and radio-resistance [21] and various systemic side effects. The drug resistance mechanisms by cancer cells attenuate the therapeutic response, leading to tumor recurrence [21]. In order, therefore, to overcome cancer drug resistance, it is necessary to use alternative therapeutic approaches; a drug combination with natural molecules represents a great strategy not only effective in overcoming cancer drug resistance but useful for increasing anti-tumor effects and reducing systemic toxicity [22].

In this regard, polyphenols, a natural compounds of plant origin, represent promising approaches for either single or combined anti-tumor therapies, displaying greater efficacy and less toxicity [23,24]. In vitro and in vivo reports, as well as human studies, corroborate polyphenols’ protective effects against neurodegenerative and inflammatory chronic diseases, obesity, diabetes [25,26,27,28] and also tumor development [29,30,31]. However, human epidemiological studies are not having much success in evaluating polyphenols’ effects due to their poor bioavailability and high metabolism rate [32]. Accumulating data showed in vitro polyphenols anti-proliferative effects on several cancer types, such as prostate [33,34], colon [35,36], breast [37,38,39], lung [40,41], bladder [42,43], pancreatic [44,45], leukemia [46,47], osteosarcoma [48,49] and ovarian [50,51], through different mechanisms of action. They can influence important molecular events related to carcinogenesis; these include the modulation of cell cycle signaling, induction of detoxifying and antioxidant enzyme systems, alteration of the epigenome and metabolism, and changes in the expression of key proteins involved in signal transduction pathways (e.g., MAPK and PI3K) or in the activity of transcription factors (e.g., NFkB) [52,53,54]. Most of the well-studied polyphenols induce apoptosis [55]. Polyphenol-mediated apoptosis occurs in some cell lines at different concentrations by directly influencing different apoptotic pathways and/or the expression of regulatory proteins. Among the molecules that exert their regulatory effect in determining cell fate are the BCL2 family members [56], the caspases [57], and the transcription factor p53 [58], which represent the most important checkpoints that control the main apoptosis polyphenol-regulated steps [55].

In this paper, we reviewed the current knowledge regarding the molecular mechanisms by which most representative polyphenol family members induce apoptotic cell death in cancer cells. This aspect is very interesting because it could contribute to the design of new therapeutic strategies against tumor involving polyphenols as adjuvants.

## 2. Chemical Structure, Sources and Pharmacokinetics of Dietary Polyphenols

**Figure 1:**
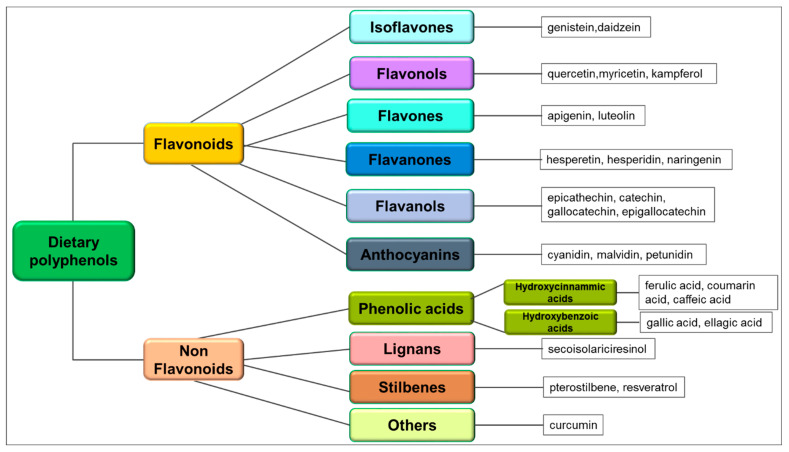
Schematic classification of dietary polyphenols and main relevant phytochemical compounds belonging to the various groups.

Polyphenols are water-soluble organic compounds; chemically, these compounds possess an aromatic ring and a benzene ring with one or more hydroxyl substituents [59]. Due to their great heterogeneity, they can be classified in two large groups, flavonoids and non-flavonoids, in turn divided into many subgroups based on the number of phenolic units within their molecular structure, substituent groups and/or the linkage type between phenolic units [59]. Isoflavones, flavonols, flavones, flavanones, flavanols and anthocyanins make up the majority of the flavonoid group [59]. Non-flavonoid compounds include phenolic acids (benzoic acid and cinnamic acid derivatives), lignans, stilbenes, and others [59] (Figure 1). Flavonoids present a C_6_-C_3_-C_6_ structure containing two benzene rings, A and B, connected by a heterocycle pyrene ring (C) that contains oxygen; most of them have a structure in which the C_2_ of the C ring is attached with a B ring, but C_3_ and C_4_ attachments are also found. Non-flavonoids, unlike flavonoids, contain only one phenolic ring [59]. It has been reported that specific chemical structural features are involved in the polyphenols’ biological actions. The structure of the catechol in the B ring is the main determinant of the radical scavenging capacity of the flavonoids; in the absence of catechol, the C_2_-C_3_ double bond, along with C_3_-OH and C_4_ carbonyl, is determinant for antioxidant action [60,61]. Moreover, the C ring chemical variations and the type and number of substituents, as well as the position of the substituents, can modify their antitumor activity. In fact, the presence of the C_2_-C_3_ double bond, the oxo group in C_4_, the open C ring and the position of the B ring (in C_2_ or C_3_) are the structural variations that not only differentiate flavones from flavanones, catechins, and isoflavones but may also affect their anticancer properties. Furthermore, both the sugar fractions and the polyhydroxylation reduce the hydrophobicity of the flavonoids by hindering their entry into the cells. In addition, the substituents’ modifications at different positions, such as at the 3’ and 4’ in the flavonoid structure, can modify their efficacy [60]. Structural modifications can be performed for the increased biological activity of the non-flavonoids. Aromatic ring modifications, changes in the hydroxyl groups position and number, and the addition of an electron-withdrawing group, as well as carboxyl group modifications, are critical structural features for the antioxidant activity of hydroxycinnamic acid [61]. Furthermore, several structures were developed from the stilbene parent molecule by hydroxyl, alkoxy, and glycoside group substitution in order to obtain derivatives with improved biological activity [61].

Flavonoids are the most widely distributed and studied polyphenols. In fact, dietary polyphenol consumption consists principally of 80% flavanols, 8% flavonols, 6% flavanones, 5% anthocyanins and less than 1% isoflavones and flavones [62].

The greatest dietary sources of flavonoids are tea, onions, apples, citrus fruits, berries, soybeans, legumes and red wine [63]. Specifically, isoflavones are very abundant compounds in legumes; in plants, they can be found mainly as inactive glycosides (e.g., daidzin and genistin) which are converted to the active corresponding aglycones (e.g., genistein, daidzein) after ingestion [64]. Flavonols (e.g., quercetin, myricetin, kampferol), although present in most edible plants (red wine, onions, blueberries and broccoli), are less concentrated than other flavonoids [65]. They mainly exist in the glycosylated form with glucose or rhamnose but can also be conjugated to galactose, xylose or arabinose [65]. Flavones, common in spices and some yellow- or orange-colored fruits and vegetables, include apigenin, luteolin, tangeritin and chrysin [66], while flavanones (e.g., hesperetin, hesperidin, naringenin) [67], generally glycosylated from a disaccharide, are the most abundant flavonoids in citrus fruits and in grapes and the medicinal herbs of Rutaceae, Rosaceae and Leguminosae. Flavanols, such as epicatechin, catechin, epicatechin gallate, epigallocatechin and epigallocatechin gallate (EGCG), are widespread in fruit and also in medicinal herbs and dietary plants (e.g., tea, apples, berries, cocoa and catechu) [68]; the oxidation of their monomeric form present in black tea following heating leads to the formation of complex molecules, such as theaflavins and thearubigins, which are dimeric and polymeric in nature, respectively [69]. Anthocyanins (e.g., cyanidin, malvidin, petunidin) are typical pigments found in red, blue or purple flowers and fruits; in particular, berries, currants, grapes and some tropical fruits have high anthocyanin content [70].

Compounds with smaller and simpler chemical structures than flavonoids belong to the non-flavonoids group, even if some of them are structurally more complex and have a high molar mass [59]. Phenolic acids, lignans, stilbenes and miscellaneous subgroups (e.g., curcuminoids) make up this group (Figure 1).

The phenolic acids include two subgroups: hydroxycinnamic and hydroxybenzoic acids. The first derive from cinnamic acid and are often present in foods as simple esters of quinic acid or glucose; the most abundant of them is the bound type, the chlorogenic acid, a combined form of caffeic acid and quinic acid, while the most common free forms are ferulic, caffeic, p-coumaric and sinapic acids [71]. Compared to the former group, the hydroxybenzoic acids (e.g., gallic, ellagic, p-hydroxybenzoic, protocatechuic, vanillic and syringic acids) are generally found in low concentrations in red fruits, black radish, mango, tea and onions [71]. Lignans (e.g., secoisolariciresinol) have 2, 3-dibenzylbutane as their basic structure, which is formed when cinnamic acid residues form a dimer [72]. Legumes, cereals in particular, wheat, pears, plums, garlic, carrots and asparagus are rich in lignans, but the main source of secoisolariciresinol are flax seeds. Stilbenes are found in small quantities in the human diet [73]; the major representative compound of this family are pterostilbene and resveratrol, which have been found in various edible natural products, such as grapes, peanuts, berries and rhubarb [73]. Curcuminoids are natural non-flavonoid polyphenol compounds derived from turmeric (*Curcuma longa*), the traditional Indian spice that is a member of the ginger family (Zingiberaceae); they include curcumin and related compounds that are bright yellow pigments constituting a group of lipophilic diketones [74].

The chemical structure, the degree of glycosylation/acylation and polymerization, the molecular size and also the solubility [75] influence polyphenol absorption and metabolism. Initially, polyphenols are conjugated with sugar moieties within plants (glycosides) and must be hydrolyzed to separate the sugar group (glycon) from the polyphenol (aglycone) prior to absorption. This process occurs in the epithelial cells of the small intestine by lactase-phlorizin hydrolase (LPH) [76]. Aglycones and simple monomeric polyphenols can be absorbed through the intestinal mucosa into the circulation, but undergo glucuronidation, sulfonoridation and methylation both within the epithelial cells and liver [77]. Since mammalians lack appropriate β-glycosidases, the glycosides cannot be absorbed; some of them can be partially absorbed due to the presence of enzyme present in the gastrointestinal microbiota. In particular, a minor dietary polyphenol amount (5–10% of total intake) can be easily absorbed using deconjugation reactions (e.g., deglycosylation) in the small intestine; these polyphenols undergo to extensive phase I (e.g., oxidation, reduction, hydrolysis, etc.) and phase II (e.g., conjugation reactions) biotransformations in enterocytes, originating water-soluble metabolites (e.g., methyl, glucuronide, sulfate derivatives, etc.) that can be rapidly released into systemic circulation for subsequent organ distribution, including to the liver [77]. Here, polyphenols undergo a further rapid phase I and II metabolism. The formation of anionic derivatives by glucuronidation and sulfatation facilitates their urinary and biliary excretion and then their rapid elimination. In the bile, some of them can be deconjugated and reabsorbed several times (enterohepatic cycle) [77]. Furthermore, 90–95% of ingested polyphenols (most flavonoids) reach the colon, where are metabolized by gut microbiota producing several metabolites of phase II (e.g., methyl, glucuronide, sulfate derivatives) or dehydroxylated, deglycosylated and demetylated products destined for faecal excretion [77].

Therefore, factors such as low water solubility, poor absorption, low tissue distribution, high rate of metabolism, inactivity of metabolic products and/or rapid elimination contribute to the low bioavailability of polyphenols, which limits their clinical applications. Several strategies have been developed over the years in order to improve the polyphenols’ pharmacokinetic profile. These include the preparation of new analogs and drug delivery systems such as emulsions and liposomes that could improve their solubility and prolong their residence time in plasma [78,79,80].

## 3. Polyphenol-Mediated Apoptosis as Anticancer Mechanism

Polyphenols are known for their beneficial properties against various pathological conditions, such as diabetes, cardiovascular and neurodegenerative diseases, as well as cancer [26,63,81]. Polyphenols’ anticancer activity is mainly attributed to their antioxidant property being strong radical scavengers, metal chelators, and modifiers of endogenous defense mechanisms, such as SOD, CAT, GPx, GSH and regulators of several proteins and transcription factors, such as Nrf2 [25,82]. Furthermore, they exhibit the ability to decrease tumor cells growth through the inhibition of polyamine biosynthesis and signal transduction enzymes like PTK, PKC and PI3K and through the expression modulation of proteins involved in cell cycle arrest, cell migration, metastasis and cell death [23,63,81,83,84,85].

**Figure 2:**
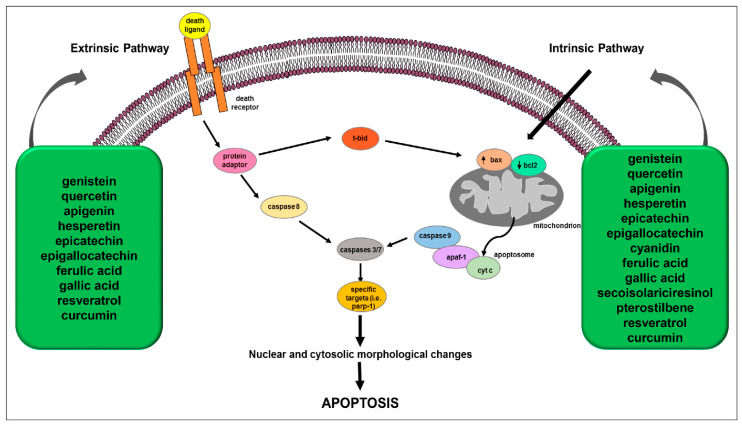
Effects of some polyphenols on extrinsic and intrinsic apoptotic pathways in cancer. ↑: increase, ↓: decrease. Adapted from Chimento, A. et al. [89].

Polyphenols’ anticancer effects largely depend on their ability to trigger apoptotic mechanisms of death [83]. At a cellular level, apoptosis can occur in mammalian cells by extrinsic or intrinsic pathways that are mainly carried out by caspases, a family of cysteine-dependent aspartate-directed proteases [86]. The extrinsic pathway is activated when a specific ligand binds (e.g., TNFα, FasL, TRAIL) its related cell surface death receptor (e.g., TNF-R, Fas, TRAIL-R); this event is followed by receptor trimerization, the recruitment of adaptors proteins (e.g., FADD) and the activation of an initiator caspase (e.g., procaspase-8) [87]. The intrinsic or mitochondrial pathway is activated by several agents, such as oxidants, toxins, drugs or ionizing radiation, which induce ROS overproduction and then oxidative stress. It involves the mitochondria and mitochondrial proteins [87], such as those belonging to the BCL2 family, which include anti-apoptotic proteins (bcl-2, bcl-xL, bcl-w, mcl-1, Bfl-1/A1), pro-apoptotic pore-formers (bax, bad, bak, bok) and (3) pro-apoptotic BH3-only proteins (bid, bik, bim, bmf, hrk, noxa, puma, etc.) [88]. The intrinsic pathway is characterized by cytochrome c translocation from the mitochondrial intermembrane space into the cytoplasm. Cytochrome c, by binding apaf-1 and procaspase-9, forms the molecular apoptosome complex, which activates caspase-9 through autocatalysis. The two apoptotic pathways can be linked by the caspase-mediated activation of some proteins (e.g., Bid) that can directly affect the mitochondrial intrinsic pathway [88,89] (Figure 2). Both extrinsic and intrinsic pathways lead to the activation of effector caspases (e.g., caspase-3) which, in turn, by targeting specific targets, cause DNA fragmentation, apoptotic bodies’ formation and finally cell death. In case of DNA damage, the activation of pro-apoptotic genes or tumor suppressor genes such as p53 can trigger the apoptosis initiation; p53 is able to modulate key control points in both intrinsic and extrinsic pathways [90] by transcriptionally upregulating apoptosis-related proteins (e.g., puma, noxa, bid and bax) expression and physically interacting with and neutralizing the anti-apoptotic activity of bcl-2 and bcl-xL; moreover, it can transactivate the death receptor genes and/or induce those (e.g., PTEN) that inhibit the antiapoptotic pathway, such as the of PI3K/AKT survival signaling [90]. In addition, massive damage to mitochondria also plays a key role in mitochondrial apoptosis [91]. Cancer cells with absent tumor suppressors’ activity may develop some survival mechanisms to prevent apoptosis progression. Tumors with loss of p53 and PTEN show high expressions of anti-apoptotic genes such as PI3K, NF-κB and BCL2. On the other hand, a reduction in the expression of pro-apoptotic genes can be expected for most tumor cells [92]. In particular, cancer cells can escape apoptosis by: (i) upregulating or downregulating the anti- or pro-apoptotic genes’ expression, respectively; (ii) modulating anti- or pro-apoptotic proteins functions through post-translational modifications (e.g., phosphorylation); or (iii) decreasing caspases function [9]. Therefore, a promising anti-tumor strategy could consist of the restoration of one or both apoptotic pathways through the inhibition of antiapoptotic factors and/or the stimulation of proapoptotic ones [10].

Several data reported that polyphenols can trigger both the extrinsic and intrinsic apoptotic pathways by inhibiting antiapoptotic factors and/or activating proapoptotic molecules; these molecular events lead to morphological and biochemical changes culminating in cancer cells’ apoptosis (Figure 2).

The main apoptotic mechanisms induced by some representative flavonoid and non-flavonoid family members in cancer cells are summarized in Table 1.

### 3.1. Isoflavones and Apoptosis

Epidemiological studies reported a lower incidence of cancer in Asian subjects who consume soy products [93]. In fact, multiple anti-cancer actions have been attributed to isoflavone genistein, the main soy product [94]. In isoflavones, the presence of –OH between C_4_ and C_7_ makes them similar to the estradiol structure. Given the structural similarity of genistein to estrogen, it may exhibit estrogenic activity by acting on estrogen receptor (ER) alpha and beta, mainly through the classical genomic mechanism [95]. However, it differs from estrogens in its preference for ERβ, showing a 20-fold higher affinity for this over ERα [96]. Evidence suggests that the metabolism and biological effects of genistein may vary with dosage or exposure levels, even depending on the cell type involved. In fact, there is a duplicity of genistein action as it appears to be a possible beneficial or therapeutic agent in some cases and an endocrine disruptor in others. In vitro studies on tumor cells highlighted very variable and contrasting biological effects, especially in relation to cell proliferation and apoptosis depending on the used dose: low doses (≤1 μg/mL) promote proliferation and inhibit apoptosis, while higher exposure levels (≥10 μg/mL) produce growth inhibitory effects and induce apoptotic mechanisms [95].

Genistein induced apoptosis in MCF-7 breast cancer cells via regulating ERα expression and altering the bax/bcl-2 ratio [97]. Bcl-2 and bcl-xL downregulation was observed in MDA-MB-231 breast cancer cells together to endogenous copper ions’ mobilization and ROS generation [98]. Recently, ROS-dependent apoptosis mediation by genistein was demonstrated in A549 and 95D non-small cell lung cancer (NSCLC) [99]. In these cell models, genistein elevated intracellular ROS generation, decreased MMP, upregulated bax expression and cytochrome c release from mitochondria and decreased bcl-2 protein levels. Further examinations revealed that the expression level of FOXO3a and puma in NSCLC was significantly increased by genistein [99]. Genistein-mediated cell apoptosis through endoplasmic reticulum stress- and mitochondria-dependent pathways was demonstrated in HL-60 leukemia cells [100]. In these cells, it promoted ROS and Ca^2+^ accumulation; decreased MMP, bcl-2 and bid protein levels; and increased the ATF-6α, GRP78, bax, bad, bak, parp-1 cleavage, caspase-9 and -3 expression [100]. Similarly, in T24 human bladder cancer cells, genistein determined apoptosis through ROS-dependent PI3K/AKT signal transduction pathway inhibition [101]. In particular, it caused mitochondrial dysfunction, which was associated with bcl-2/bax expression ratio decrease; parp-1 cleavage; cytochrome c release into cytosol; and caspase-3, -8 and -9 activation [101]. Genistein prompted the generation of significant ROS amounts favoring cell death in the HepG2 liver cancer cell line [102]. In this cell model, mitochondrial apoptosis was associated with cytosolic cytochrome c, bax, cleaved caspase-3 and -9 expression increase and bcl-2 decrease [102]. Moreover, genistein-mediated apoptosis was demonstrated in HT-29 colon cancer cells where it activated caspase-3 [103]; the same effects on caspase-3 activity together with a bax expression increase was observed in HCT-116 and LoVo colon cancer cells [104]. Similarly, bcl-2, bcl-xL, c-IAP1, survivin and NF-κB downregulation after genistein use was found in A2780 and C200 ovarian cancer cells [105]. On the contrary, in LNCaP tumor prostate cells, genistein induced apoptosis via the TRAIL-mediated disruption of MMP [106]. In the same tumor cell model and C4-2B bone metastatic LNCaP-derivative prostate cancer cells, genistein reduced cell proliferation and induced apoptosis, as confirmed by elevated DNA fragmentation; moreover, the combination of low dose of genistein and daidzein displayed synergistic preventive effects on prostate cancer cells when compared with single soy isoflavone [107].

### 3.2. Flavonols and Apoptosis

Quercetin, the most predominant flavonol in plants, possesses phytoestrogenic action, as it is structurally similar to estradiol. Several studies demonstrated its anti-cancer actions involving growth inhibition and apoptosis induction through the modulation of multiple signaling pathways including those of p53, NF-κB, MAPK, JAK/STAT, PI3K/AKT, and Wnt/β-catenin; in addition, quercetin controls the activity of oncogenic and tumor suppressor ncRNAs [108,109]. In particular, quercetin induced intrinsic apoptotic mechanism via bax increase, bcl-2 decrease [110,111], MMP decrease, AIF release and caspase-6, -9 activation in MCF-7 breast cancer cells [111]. Quercetin-mediated mitochondrial apoptotic effects were also observed in HL-60 leukemia cells through COX2 inhibition, caspase-3 activation, bax and bad increase, bcl-2 decrease, cytochrome c release and parp-1 cleavage [112]. Moreover, quercetin showed an inhibitory effect on A549 lung cancer cell proliferation, by inducing apoptosis through BAX and BCL2 gene expression modulation and caspase-3 activation [113]. Zhou and coworkers demonstrated that quercetin-mediated apoptosis in SKOV-3 and A2780 human ovarian cancer cell lines was dependent on miRNA regulation; quercetin, by stimulating miR-145 expression, induced caspase-3 expression and then apoptosis [114]. The combinatory treatment of quercetin and irradiation caused DNA damage and apoptosis induction in OV2008 and A2780 ovarian cancer cells; in particular, the cell death occurred by bax and p21 increase and bcl-2 decrease in protein expression, in a p53 dependent manner [115]. On the contrary, quercetin triggered a caspase-dependent extrinsic apoptosis by upregulating caspase-8 and -3 and parp-1 cleaved forms in BT474 human breast cancer cells [116]. A study performed in Hela cervical cancer cells demonstrated that the genes involved in the apoptotic extrinsic pathway are upregulated by quercetin [117]. In particular, the expression of TRAIL, FasL, TNF and their receptors (Fas, TNFSF10, TNFRSF10A, TNFRSF10B, TNFRSF1A, TNFRSF1B, TNFRSF21, TNFRSF25), as well as those of TRADD, CRADD and DEDD, was elevated after quercetin treatment. Moreover, the enhanced expression of caspase-8, -10, -3 and -7 confirmed the role of the extrinsic pathway in quercetin-mediated apoptosis [117].

### 3.3. Flavones and Apoptosis

Apigenin, a member of the flavone family, is a nutraceutical that has been reported to be an anticancer agent in several experimental studies. It is able to reduce cell growth and induce apoptosis in different tumors such as skin, colon, prostate, breast, lung, liver, pancreas, cervical, oral and stomach [118,119]. Apigenin triggers both the intrinsic and extrinsic pathways of apoptosis. The apigenin-mediated intrinsic pathway occurs primarily via cytochrome c release, bax expression increase and caspase-3 activation, while the extrinsic pathway activation is confirmed by caspase-3, caspase-8 and TNF-α mRNA upregulation [118]. The pro-apoptotic properties of apigenin was demonstrated in PC-3 and DU145 prostate cancer cells. In these cell models, it triggered apoptosis by both bax protein expression and cytochrome c release increase, and XIAP, c-IAP1, c-IAP2, survivin, bcl-2 and bcl-xL decrease. In particular, the apigenin-mediated increase of bax was due to the dissociation of bax from Ku70, an important event for bax apoptotic activity [120]. The same effects on BCL2 family members was found in the T24 bladder cancer cell line, where it upregulated bax, bad and bak and downregulated bcl-2, bcl-xL and mcl-1 protein expression; these events were associated with caspase-9, -3, -7 activation, cytochrome c release and parp-1 cleavage [121]. Woo and coworkers demonstrated that apigenin was able to induce apoptosis by regulating AKT and MAPK pathways in A375SM human melanoma cells. In particular, in apigenin-treated cells, p-ERK and p-JNK increased, while p-p38 and p-AKT protein expression levels decreased; these molecular events correlated to downregulation of bcl-2, and upregulation of parp-1 and caspase-9 cleaved forms, as well as bax and p53, in a dose-dependent manner [122]. The apigenin-mediated extrinsic apoptotic mechanism was demonstrated in SCC25 and A431 HNSCC cells via TNF-R, TRAIL-R and bcl-2-mediated caspase-dependent cell death [123]. Additionally, apigenin-induced apoptosis was related to p53 expression, bax/bcl-2 ratio increase, caspases-9 and -8 activation and parp-1 cleavage in malignant mesothelioma cells [124].

### 3.4. Flavanones and Apoptosis

Among the flavanones, hesperetin showed great anti-tumor efficacy that primarily occurs via targeting apoptosis-mediated cell death pathways [125]. A study performed in both an in vitro and in vivo gastric cancer cell model demonstrated that hesperetin induced the intrinsic apoptotic mechanism by increasing ROS levels; in particular, it determined apaf-1 and bax expression upregulation, caspases-3 and -9 activation, MMP and bcl-2 expression decrease, cytochrome c release and DNA fragmentation [126]. The same ROS-dependent apoptotic mechanism was also observed in Eca109 esophageal [127], HT-29 colon [128] and MCF-7 breast [129] cancer cells. In Eca109 cells, apoptosis was confirmed by caspase-9 and -3 activation; apaf-1, SuFu and bax protein expression increase; and bcl-2 decrease [127]. In HT-29 cells, hesperetin-mediated apoptosis was associated with cytochrome c release, bax and cleaved caspase-3 expression increase and bcl-2 expression decrease, as well as with ROS increase and antioxidants enzymes such as SOD, CAT and GPx inhibition [128]. In MCF-7 cells, hesperetin treatment caused phosphatidyl-serine externalization, DNA fragmentation, caspase-9 and -7 activation, MMP loss, cytochrome c release, bax/bcl-2 ratio increase and parp-1 cleavage [129]. Conversely, in H522 lung cancer cells, hesperetin extrinsically determined apoptosis in a p53- and bax-independent manner through Fas and FADD overexpression and caspase-8 activation [130]. In non-small cell lung cancer, the A549 hesperetin-mediated suppression of Hsp70, a negative regulator of the mitochondrial apoptosis pathway, was associated with reduced cytosolic bax and increased mitochondrial bax levels, leading to mitochondrial apoptotic cascade activation [131]. Moreover, the hesperetin-mediated both intrinsic and extrinsic apoptotic pathway was found in SiHa cervical cancer cell lines [132].

### 3.5. Flavanols and Apoptosis

Epicatechin, a flavanol derived primarily from green tea and other plants, is found in most commonly consumed food beverages, unlike many phytochemicals. Several studies suggested that one of the possible mechanisms of epicathechin-mediated cancer inhibition is apoptosis induction [133]. The apoptotic effects of epicatechin were evaluated in breast cancer cells. In MDA-MB-231 cells, it upregulated death receptor (DR4/DR5), increased ROS production and mitochondrial permeability and modulated pro-apoptotic proteins (e.g., cytochrome c, Smac/Diablo, and HtrA2/Omi) [134]. In MCF-7 cells, it did not generate changes in the TRAIL receptor, but an increase in ROS and the upregulation of pro-apoptotic proteins (e.g., bad and bax) were observed [134]. In another cancer cell model, SW480 human colon cells, the epicathechin apoptotic effects were related to bax and p53 mRNA and protein expression increase, as well as to bcl-2 decrease and DNA fragmentation [135]. Moreover, a recent study demonstrated that epicatechin acts as an agonist towards the novel androgen receptor ZIP9 by miming testosterone-induced apoptosis through this receptor in prostate and breast cancer cells; in particular, it exerted androgenic actions inducing cell death through an intrinsic apoptotic pathway, as confirmed by bax expression increase, cytochrome c release and caspase-3 activation [136].

### 3.6. Anthocyanins and Apoptosis

Several studies indicated anthocyanins as nutraceutical for cancer prevention and management. Anthocyanins’ anti-tumor actions are due to their antioxidant, anti-inflammatory and anti-proliferative properties and their ability to regulate apoptosis-related mediators, including p53, bcl-2, bax, cytochrome c and caspase-3 in several cancer cell models [137]. In DU145 and LnCap prostate cell lines, cyanidin determined caspase-3 activation and DNA fragmentation [138], while in U87 glioblastoma cells, apoptosis occurred via bax and p53 mRNA expression increase and bcl-2 decrease [139]. Another recent in vitro study demonstrated that cyanidin induced apoptosis through the upregulation of PPARγ, p21 and bax mRNA expression in osteosarcoma cell lines [140].

### 3.7. Phenolic Acids and Apoptosis

Phenolic acids are a subgroup of non-flavonoid compounds that are associated with potent anticancer abilities demonstrated in several in vitro and in vivo studies. They act as anti-tumor agents by promoting apoptosis, reducing proliferation and differentiation and targeting other hallmark of cancer, such as angiogenesis and metastasis [141].

The hydroxycinnamic derivative ferulic acid was able to trigger apoptosis process. Ferulic acid-mediated apoptosis was evaluated in MCF-7 breast cancer cells where it caused both caspase-8 and -9 activation [142]. The same caspases’ activation was found in HepG2 hepatic cancer cells after ferulic acid treatment, although caspase-8 activation was more involved [142]. Furthermore, recently, it has been demonstrated that ferulic acid increased bax/bcl-2 ratio and displayed a synergistic activity with epirubicin on apoptosis induction in MDA-MB-231 breast cancer cells [143]. Additionally, ferulic acid treatment remarkably downregulated the PI3K/AKT, reduced bcl-2 and mcl-1 expression and increased that of bax in Caski cervical cancer cells [144].

Similarly, gallic acid was able to induce apoptosis through Fas, FasL and DR5 expression upregulation; caspase-8, -9 and -3 activation; bad and bak protein expression increase; and bcl-2 decrease in AGS gastric cancer cells [145]. In particular, in these cells, p53 was shown to be involved in gallic-acid-mediated apoptosis [145]. Additionally, in MDA-MB-231 breast cancer cells, it increased caspase-8 and -9 activity, decreased MMP and induced cytochrome c release [146]. The ability of gallic acid to trigger apoptosis and also potentiate the apoptotic effect of paclitaxel and carboplatin has also been demonstrated in MCF-7 breast cancer cells where it caused the overexpression of bax, caspase 3 and p53 and the downregulation of bcl-2 [147].

### 3.8. Lignans and Apoptosis

Many studies demonstrated that natural lignans possess great anti-tumor activities against a variety of human cancer cell lines by inhibiting all phases of carcinogenesis, including initiation, promotion and progression and by modulating different signaling pathways involved in cell proliferation and death control [148]. Dietary lignan secoisolariciresinol diglucoside can mainly induce the intrinsic apoptotic mechanism by targeting several molecules. Recently, its apoptotic effects were investigated in both 2D and 3D cultures of SW480 colon cancer cells. Results indicated a significant increase in AIF and caspase-3 staining and gene expression in lignan-treated cells compared to the control group in both monolayer and spheroid culture cells [149]. Moreover, a mechanistic study showed that the same compound reduced MCF-7 cell proliferation via downregulating ER and growth factor-mediated signaling, also triggering apoptosis. In particular, it significantly decreased the mRNA expression of bcl-2, cyclin D1, pS2, ERα, ERβ, EGFR and IGF-IR [150].

### 3.9. Stilbenes and Apoptosis

Stilbenes displayed an extraordinary potential for the prevent and treatment of different diseases, including cancer, due to their antioxidant and anti-inflammatory properties and ability to induce cell death [151]. Pterorostilbene is an active apoptotic agent in different in vitro cell models. Liu et al. reported its ability to inhibit osteosarcoma cell growth via JAK2/STAT3 signaling pathway downregulation. This led to a decrease in the protein expression of antiapoptotic factors (e.g., bcl-xL and mcl-1) and an increase in that of proapoptotic proteins (e.g., bax, bak, cytochrome c, caspase-3) and cyclin-dependent kinase inhibitors (e.g., p21 and p27) [152]. Pterostilbene treatment inhibited MCF-7 and MDA-MB-231 breast cancer growth in vitro through caspase-3, -7-dependent apoptosis; indeed, it decreased MMP and increased superoxide anions, activating downstream effector caspases [153]. In a recent work, by examining pterostilbene effects in the mutant p53-breast cancer cell lines MDA-MB-231 and T-47D, it has been demonstrated that it decreased mutant p53 protein expression while increasing the pro-apoptotic bax protein expression [154]. The same effect on bax expression was observed in MDA-MB-468 breast cancer cells, where it inhibited AKT and mTOR phosphorylation [155]. Another investigation demonstrated that this compound activated caspase-3 and decreased the expression of cell survival proteins bcl-2 and bcl-xL in HEC-1A and ECC-1 endometrial cells. Interestingly, it increased the anti-tumor activity of megestrol acetate, a commonly used drug in endometrial cancer patients [156]. The pro-apoptotic effects of pterostilbene were demonstrated in HeLa cervical cancer cells through the ROS-mediated activation of caspase-3 and caspase-9, as well as the downregulation of the antiapoptotic proteins bcl-2 and bcl-xL [157]. The proposed mechanism for ROS-dependent pterostilbene anticancer activity was assessed using HepG2 liver cancer cells; in these cells, it increased p53 expression, which, in turn, decreased SOD2 expression and induced ROS generation, leading to apoptosis [158].

### 3.10. Curcuminoids and Apoptosis

Polyphenolic curcuminoids, including the curcumin (or curcumin I), demethoxy curcumin (or curcumin II) and bis-demethoxycurcumin (curcumin III), represent 95% of the standardized extract from the underground root (rhizome) of *Curcuma longa* L. [159]. Curcumin, the most abundant curcumonoid (~77%), displays anticancer effects that occur by interfering with various cellular pathways and inducing/inhibiting the expression of several proteins related to proliferation, angiogenesis, invasion, migration, metastasis and apoptosis [78,160]. Curcumin-mediated apoptosis in cancer cells occurs by modulating both extrinsic and intrinsic apoptotic pathways mediators. Extrinsic apoptosis has been demonstrated through increased ROS production in leukemia [161], melanoma [162], breast [163] and renal cancer cells [164]. Furthermore, the upregulation of TRAIL, in glioblastoma multiforme [165], renal [164], breast [163], prostate cancer cells [166] and FasL in hepatocellular carcinoma [167] plays a key role in curcumin-mediated extrinsic apoptosis. In a recent paper, Obaidi and co-workers demonstrated that curcumin sensitized cancerous kidney cells to TRAIL-induced apoptosis via the Let-7C mediated deregulation of cell cycle proteins and cellular metabolism [168]. On the other hand, changes in mitochondria function, as well as the p53-dependent bax upregulation in breast [169] and colon cancer cells [36] and the bcl-2 down-regulation in laryngeal [170], breast [171] and prostate [172] cancer cells, are the most crucial changes that occur in curcumin-mediated intrinsic apoptosis. Furthermore, the ability of curcumin to activate caspase-8 in melanoma [173], and caspase-3 in hepatocellular carcinoma [167], neuroblastoma [174], linfoma [175] and lung cancer cells [176] confirmed its pro-apoptotic role. In RT4 schwannoma cells curcumin reduced bcl-2 expression, enhanced that of bax, and activated caspase-3, caspase-9, and parp-1 via miRNA 344a-3p upregulation [177]. Recently, apoptotic cell death by curcumin was observed in BCPAP and TPC-1 papillary thyroid cancer cells and derived thyroid cancer stem-like cells (thyrospheres) by targeting JAK/STAT3 signaling pathway. In particular, it suppressed the constitutive JAK/STAT3 signaling pathway and upregulated the ROS level to induce apoptosis. Indeed, protein expression analysis showed that curcumin-mediated caspase-3 activation, bcl-2 and bcl-xL decrease and bax increase were markedly reversed by NAC, a known ROS scavenger; these results confirmed the role of oxidative stress in curcumin-induced apoptosis [178].

**Table 1:** Apoptotic mechanisms induced by some representative flavonoid and non-flavonoid family members in cancer. ↓: decrease, ↑: increase; NSCL: non-small cell lung cancer; HNSCC: head and neck squamous cell carcinoma.

Family	Phytochemical Compounds	Types or Names of Cancer	Mechanism Leading to Apoptosis	References
Flavonoids				
Isoflavones	genistein	breast	bax↑, bcl-2↓	[97]
			bcl-2↓, bcl-xL↓, ROS↑	[98]
		NSCL	ROS↑, MMP↓, cytochrome c↑, bcl-2↓, bax↑, FOXO3A↑, PUMA↑	[99]
		leukemia	ROS↑, MMP↓, bcl-2↓, bid↓, ATF-6α↑, GRP78↑, bax↑, bad↑, bak↑, cleaved parp-1↑, caspase-9↑, caspase-3↑	[100]
		bladder	bcl-2/bax ratio↓, caspase-3, -8 and -9 activation, cytochrome c↑, cleaved parp-1↑	[101]
		liver	ROS↑, cytochrome c↑, Bax↑, cleaved caspase-3↑, cleaved caspase-9↑, bcl-2↓	[102]
		colon	caspase-3 activation	[103]
			caspase-3 activation, bax↑	[104]
		ovarian	bcl-2↓, bcl-xL↓, c-IAP↓, survivin↓	[105]
		prostate	TRIAL↑, MMP↓	[106]
			DNA fragmentation↑	[107]
Flavonols	quercetin	breast	bax↑, bcl-2↓	[110,111]
			AIF↑, MMP↓, caspase-6, -9 activation	[111]
			caspase-8, -3 activation, cleaved parp-1↑	[116]
		leukemia	bax↑, bad↑, bcl-2↓, cytochrome c↑, caspase-3 activation, cleaved parp-1↑	[112]
		lung	bax↑, bcl-2↑, caspase-3 activation	[113]
		ovarian	caspase-3 activation	[114]
			bax↑, p21↑, bcl-2↓, p53↑	[115]
		cervical	TRAIL↑, FasL↑, TNF↑, Fas↑, TNFSF10↑, TNFRSF10A↑, TNFRSF10B↑, TNFRSF1A↑, TNFRSF1B↑, TNFRSF21↑, TNFRSF25↑, TRADD↑, CRADD↑, DEDD↑, caspase-8↑, -10↑, -3↑, -7↑	[117]
Flavones	apigenin	prostate	bax↑, bcl-2↓, bcl-xL↓, c-IAP1↓, c-IAP2↓, XIAP↓, survivin↓, cytochrome c↑	[120]
		bladder	bax↑, bad↑, bak↑, bcl-2↓, bcl-xL↓, mcl-1↓, caspase-9, -3, -7 activation, cytochrome c↑, cleaved parp-1↑	[121]
		melanoma	bcl-2↓, bax↑, cleaved caspase-9 ↑, cleaved parp-1↑, p53 ↑	[122]
		HNSCC	TNF-R↑, TRAIL-R↑, bcl-2↓, caspase activation	[123]
		mesothelioma	bax/bcl-2 ratio↑, p53↑, caspase-8, -9 activation, cleaved parp-1↑	[124]
Flavanones	hesperetin	gastric	bax↑, apaf-1↑, bcl-2↓, ROS↑, caspase-9, -3 activation, MMP↓, cytochrome c↑, DNA fragmentation	[126]
		esophageal	ROS↑, bax↑, apaf-1↑, SuFu↑, bcl-2↓, caspase-9, -3 activation	[127]
		colon	ROS↑, bax↑, bcl-2↓, cytochrome c↑, cleaved caspase-3 ↑, SOD↓, CAT↓, GPx↓	[128]
		breast	ROS↑, bax/bcl-2 ratio↑, phosphatydilserine externalization, caspase-9, -7 activation, MMP↓, cytochrome c↑,cleaved parp-1↑, DNA fragmentation	[129]
		lung	Fas↑, FADD↑, caspase-8 activation	[130]
			cytosolic bax↓, mitochondrial bax↑	[131]
		cervical	extrinsic and intrinsic apoptotic pathways	[132]
Flavanols	epicatechin	breast	DR4↑, DR5↑, ROS↑, MMP↓, cytochrome c↑, Smac/Diablo↑, HtrA2/Omi↑, TRAIL↑, bad↑, bax ↑	[134]
			bax↑, cytochrome c↑, caspase-3 activation	[136]
		colon	bcl-2↓, bax↑, p53↑, DNA fragmentation	[135]
		prostate	bax↑, cytochrome c↑, caspase-3 activation	[136]
Anthocyanins	cyanidin	prostate	caspase-3 activation, DNA fragmentation	[138]
		glioblastoma	bax↑, p53↑, bcl-2↓	[139]
		osteosarcoma	bax↑, PPARγ↑, p21↑	[140]
**Non Flavonoids**				
Hydroxybenzoic acids	ferulic acid	breast	caspase-8, -9 activation	[142]
			bax/bcl-2 ratio↑	[143]
		hepatic	caspase-8, -9 activation	[142]
		cervical	bax↑, bcl-2↓, mcl-1↓	[144]
Hydroxycinnamic acids	gallic acid	gastric	Fas↑, FasL↑, DR5↑, caspase-8, -9, -3 activation, bad↑, bak↑, bcl-2↓, p53↑	[145]
		breast	MMP↓, caspase-8, -9 activation, cytochrome c↑	[146]
			bax↑, bcl-2↓, caspase-3 ↑, p53↑	[147]
Lignans	secoisolariciresinol	colon	AIF↑, caspase-3↑	[149]
		breast	bcl-2↓	[150]
Stilbenes	pterostilbene	osteosarcoma	bax↑, bak↑, bcl-xL↓, mcl-1↓, caspase-3 ↑, cytochrome c↑	[152]
		breast	superoxide anion↑, MMP↓, caspase-3, -7 activation	[153]
			bax↑, p53 mutant↓	[154]
			bax↑	[155]
		endometrial	bcl-2↓, bcl-xL↓, caspase-3 activation	[156]
		cervical	ROS↑, bcl-2↓, bcl-xL↓, caspase-9, -3 activation	[157]
		liver	ROS↑, SOD2↓, p53↑	[158]
Curcuminoids	curcumin	leukemia	ROS↑	[161]
		melanoma	ROS↑	[162]
			caspase-8 activation	[173]
		breast	ROS↑, TRAIL↑	[163]
			p53↑, bax↑	[169]
			bcl-2↓	[171]
		renal	ROS↑, TRAIL↑	[164]
			TRAIL↑	[168]
		glioblastoma	TRAIL↑	[165]
		prostate	TRAIL↑	[166]
			bcl-2↓	[172]
		hepatocellular carcinoma	FasL↑ caspase-3 activation	[167]
		laryngeal	bcl-2↓	[170]
		colon	p53↑, bax↑	[36]
		neuroblastoma	caspase-3 activation	[174]
		linfoma	caspase-3 activation	[175]
		lung	caspase-3 activation	[176]
		schwannoma	bcl-2↓, bax↑, parp-1↑, caspase-3↑, caspase-9↑	[177]
		thyroid	ROS↑, caspase-3 activation, bcl-2↓, bcl-xL↓, bax↑	[178]

## 4. Dietary Polyphenols and Drug Synergism in Cancer Therapy

In order to increase the chemopreventive effects of polyphenols, they are often combined with conventional chemotherapy drugs or even with other dietary polyphenols [179,180]. These associations enhance the efficacy of several chemotherapy agents or potentiate the anticancer activity of individual combined natural compounds [179,180] (Table 2). In particular, the improved anti-tumor activity deriving from pharmacological synergy with cytotoxic agents is associated with a lower chemotherapy toxicity in normal tissues, which could be due to an improvement of its pharmacokinetic and metabolic characteristics [179,180].

Genistein enhances the anticancer effect of cisplatin in CaSki cervical cancer cells [181]. The combination of genistein and cisplatin in these cells inhibited ERK1/2 phosphorylation, decreased bcl-2 protein expression levels and increased those of p53 and cleaved caspase-3 [181]. Moreover, it synergistically improved the anti-tumor action of antineoplastic centchroman (CC) in MCF-7 and MDA-MB-231 breast cancer cells [182]; this occurred by cell cycle arrest at the G2/M phase that culminated in ROS-dependent apoptosis. In particular, their combination determined bax and bcl-2 ratio dysregulation and mitochondrial dysfunction, together with caspase-3, -7 and -9 activation and parp-1 cleavage. Further, genistein and CC significantly inhibited PI3K/AKT/NF-κB phosphorylation, thus enhancing apoptosis [182].

Cisplatin cytotoxicity can be also enhanced by quercetin in HeLa and SiHa human cervical cancer cells [183]. Quercetin inhibited HeLa and SiHa cell viability in a dose- and time-dependent manner, and its combination with cisplatin had stronger cytotoxic effects than their individual effects. Besides, quercetin combined with cisplatin group induced more cell apoptosis in contrast to single treatment [183]. A similar drug synergism was also responsible for enhanced methotrexate chemotherapeutic efficacy against Saos-2 osteosarcoma [184]. The analysis of mRNA expression outcomes indicated that the combination therapy significantly upregulated p53, CBX7 and CYLD tumor suppressor genes and reduced anti-apoptotic genes bcl-2 and miR-223, which can lead to proliferation inhibition and apoptosis induction [184]. Moreover, a recent study demonstrated that quercetin, in combination with tamoxifen, controlled several apoptosis-related gene expression, including those of p53, bax, p21 and bcl-2 in MCF-7 breast cancer cells, leading to apoptosis regulation [185].

Studies performed on HONE1 and CNE2 human nasopharyngeal carcinoma cell lines revealed that apigenin significantly enhanced anti-tumor capacity of cetuximab by suppressing EGFR signaling in vivo and in vitro [186]. Results demonstrated that their combination could better suppress the viability and induce pro-apoptotic effects. In fact, the elevated bax/bcl-2 ratio and caspase-3 expression, as well as the decreased p-EGFR, p-AKT, p-STAT3 and cyclin D1 protein levels mediated by chemotherapy agent, were remarkably enhanced by apigenin in both cell lines [186]. Another work showed that apigenin, when used with cisplatin, reduced the proliferation of A549 lung, MCF-7 breast, HCT 116 colorectal, and HeLa cervical cancer cells; this effect was associated with increased cisplatin-induced DNA damage and apoptosis in a p53-dependent manner [187]. The combinatory cytotoxic effects of apigenin and paclitaxel was evaluated in HeLa cervical, A549 lung, Hep3B negroid hepatocyte and HEK293A embryonic kidney cancer cell lines [188]. Results indicated that combined treatment significantly enhanced paclitaxel cytotoxicity in all human cancer cells tested. Moreover, the ROS-mediated activation of caspase-2 and MMP decrease were essential for apigenin-/paclitaxel-induced apoptosis in HeLa cells [188]. In Jurkat lymphoid and THP-1 myeloid leukaemia cell lines, apigenin, in combination with etoposide or cyclophosphamide, induced apoptosis via the mitochondrial pathway by increasing cytochrome c, Smac/Diablo and HtrA2/Omi expression and by caspase-9 and -3 activation [189]. Moreover, the effects of several polyphenols (apigenin, quercetin, emodin and rhein) alone and in combination with three alkylating agents (cisplatin, cyclophosphamide and chlorambucil) were evaluated in Jurkat and CCRF-CEM lymphoid and THP1 and KG1a myeloid leukaemia cells lines [190]. In lymphoid leukaemia cell lines, a synergistic reduction in ATP and glutathione levels, a cell cycle arrest, DNA damage and apoptosis induction were observed by using quercetin, apigenin, emodin and rhein in combination with cisplatin and cyclophosphamide; the same effects are produced using apigenin and rhein with chlorambucil [190]. In myeloid leukemia cells, the combined use of apigenin, quercetin and emodin showed similar synergistic effects with all alkylating agents [190]. He and co-workers demonstrated that hesperetin was able to potentiate cisplatin-mediated apoptosis in HGC-27, SGC-7901 and MGC-803 human gastric cell lines [191]. In particular, this drug combination remarkably increased the expression of PTEN, cytosolic cytochrome c, bax, cleaved caspase-9 and -3 and AIF; moreover, p-AKT and bcl-2 protein expression levels were reduced [191]. The apoptosis- and oxidative-stress-related effects of cyanidin-3-O-glucoside, cisplatin and their combination were investigated on HeLa cervical cancer cells [192]. Results showed that cyanidin-3-O-glucoside and cisplatin increased oxidative stress by downregulating Nrf2 and consequently HO-1 and NQO1 expression. Furthermore, this combination promoted cell apoptosis by increasing the mRNA expression ratio of bax/bcl-2 [192]. The anti-tumor effects of cisplatin were also increased by gallic acid in A549 human NSCLC [193]. They were dependent on JAK/STAT3 signaling pathway modulation and changes in the expression of downstream apoptotic molecules, such as bax upregulation and bcl-2 downregulation [193].

Other studies determined the potential of pterostilbene to enhance gemcitabine tumor cytotoxicity and chemosensitivity in MIA PaCa-2 and MIA PaCa-2 ^GEMR^ (gemcitabine-resistant) pancreatic ductal adenocarcinoma cells [194]. In these cells, pterostilbene induced S-phase cell cycle arrest, apoptosis and autophagic cell death and inhibited multidrug resistance protein 1 (MDR1) expression by downregulating RAGE/PI3K/AKT signaling in both cell lines [194]. Another combination of pterostilbene and EGCG showed additive antiproliferative effects in MIA PaCa-2 and PANC-1 pancreatic cancer cell lines [195]. This combination induced apoptosis by mitochondrial depolarization and cytochrome c release increase in MIA PaCa-2 but not in PANC-1 cells. Moreover, while EGCG alone increased caspase-3/7 activity in MIA PaCa-2 cells, the same association did not significantly cause this effect in either cell line [195].

Several reports demonstrated the synergistic effects of several chemotherapeutic drugs in combination with curcumin. Curcumin combined with 5-fluorouracil (5-FU) or doxorubicin (DOX) chemosensitized the NT8e HNSCC cells towards apoptosis and promoted cell proliferation inhibition [196]. In particular, these combinations reduced cell proliferation through EGFR/ERK1/2 signaling downregulation and caused cell cycle arrest at the G1/S phase; moreover, apoptosis occurred by inhibiting bcl-2, increasing bax and cleaved parp-1 expression and activating caspase-3 [196]. Therefore, in MCF-7 breast cancer cells, the simultaneous treatment of paclitaxel with curcumin exhibited synergistic growth inhibition through EGFR signaling modulation and induced significant apoptosis as demonstrated by bax increase and bcl-2 protein expression decrease [197]. Cell death induced by curcumin and paclitaxel was also evaluated in both MCF-7 and MDA-MB-231 human breast cancer cell lines [198]. Results confirmed that this combination resulted in apoptosis induction as evidenced by caspase-3 and cleaved parp-1 expression increase; these effects were more evident when curcumin was used in combination than when used alone [198]. The ability of curcumin to improve the paclitaxel-induced apoptosis was also demonstrated in CaSki and HeLa human cervical cancer cell lines wherein the anti-tumor effects were associated with the NF-κB signaling inhibition together with p53 and caspase-3 expression increase [199]. Recently, a synergistic induction of apoptosis by quercetin and curcumin was observed in K562 chronic myeloid leukemia cells [200]. Gene expression analysis revealed that their combination was effective on genes that were particularly related to p53, NF-κB, AKT1, FasL and TGF-α pathways; in particular CDKN1B, AKT1 and IFN-γ are downregulated, while BTG2, p21, p27, CDKN1A, Fas and FasL are upregulated in this cell model [200].

**Table 2:** Anti-cancer effects of the dietary polyphenol and/or chemotherapy drug combination. ↑: increase, ↓: decrease.

Phytochemical Compounds/ Chemotherapy Drugs	Cell Lines	Outcomes	References
genistein+cisplatin	CaSki cervical cancer	ERK1/2 phosphorylation↓, bcl-2↓, p53↑, cleaved caspase-3↑	[181]
genistein+centchroman	MCF-7, MDA-MB-231 breast cancer	PI3K/AKT/NF-κB phosphorylation↓, cell cycle arrest at G2/M phase, ROS↑, bax↑, bcl-2↓, caspase-3, -7, -9↑, cleaved parp-1↑	[182]
quercetin+cisplatin	HeLa, SiHa cervical cancer	cell viability↓, apoptosis↑	[183]
quercetin+methotrexate	Saos-2 osteosarcoma	cell viability↓, apoptosis↑, p53↑, CBX7↑, CYLD ↑, bcl-2↓, miR-223↓	[184]
quercetin+tamoxifen	MCF-7 breast cancer	apoptosis↑, p53↑, bax↑, p21↑, and bcl-2↓	[185]
apigenin+cetuximab	HONE1, CNE2 nasopharyngeal carcinoma	cell viability↓, apoptosis↑, bax/bcl-2 ratio↑, caspase-3↑, p-EGFR↓, p-AKT↓, p-STAT3↓, cyclin D1↓	[186]
apigenin+cisplatin	A549 lung, MCF-7 breast, HCT 116 colorectal, HeLa cervical cancers	cell viability↓, apoptosis↑, p53↑, DNA damage↑	[187]
apigenin+paclitaxel	HeLa cervical, A549 lung, Hep3B negroid hepatocyte, HEK293A embryonic kidney cancer	cell viability↓	[188]
	HeLa cervical cancer	apoptosis↑, ROS↑, caspase-2↑, MMP ↓	[188]
apigenin+etoposide apigenin+cyclophosphamide	Jurkat lymphoid and THP-1 myeloid leukaemia	apoptosis↑, cytochrome c↑, Smac/Diablo↑, HtrA2/Omi ↑, caspase-9, -3 activation	[189]
apigenin+quercetin+emodin+rhein+ cisplatin and cyclophosphamide apigenin+rhein+ chlorambucil	Jurkat and CCRF-CEM lymphoid leukaemia	apoptosis↑, ATP↓, glutathione↓, cell cycle arrest, DNA damage↑	[190]
apigenin+quercetin,+emodin+cisplatin, cyclophosphamide and chlorambucil	THP1 and KG1a myeloid leukaemia	apoptosis↑, ATP↓, glutathione↓, cell cycle arrest, DNA damage↑	[190]
hesperetin+cisplatin	HGC-27, SGC-7901, and MGC-803 gastric	PTEN↑, cytosolic cytochrome c↑, bax↑, cleaved caspase-9 and -3↑, AIF↑, p-AKT↓, bcl-2↓	[191]
cyanidin-3-O-glucoside+ cisplatin	HeLa cervical cancer	Nrf2↓, HO-1↓, NQO1↓, bax/bcl-2 ratio↑	[192]
gallic acid+cisplatin	A549 non-small cell lung cancer	JAK/STAT3↓, bax↑, bcl-2↓	[193]
pterostilbene+gemcitabine	MIA PaCa-2, MIA PaCa-2 GEMR (gemcitabine-resistant) pancreatic ductal adenocarcinoma	S-phase cell cycle arrest, apoptosis↑, MDR1↓, RAGE/PI3K/AKT signaling↓	[194]
pterostilbene+epigallocatechin gallate	MIA PaCa-2 pancreatic cancer	cell proliferation↓, mitochondrial depolarization, cytochrome c release↑	[195]
	PANC-1 pancreatic cancer	cell proliferation↓	[195]
curcumin+5-fluorouracil or doxorubicin	NT8e head and neck squamous cell carcinoma	cell proliferation↓, EGFR/ERK1/2 signaling↓, G1/S cell cycle arrest, apoptosis↑, bcl-2↓, bax↑, caspase-3 activation, cleaved parp-1↑	[196]
curcumin+paclitaxel	MCF-7 breast cancer	cell proliferation↓, EGFR signaling↓, apoptosis↑, bcl-2↓, bax↑	[197]
curcumin+paclitaxel	MCF-7, MDA-MB-231 breast cancer	apoptosis↑, caspase-3↑, cleaved parp-1↑	[198]
curcumin+paclitaxel	CaSki, HeLa cervical cancer	NF-κB signaling↓, p53↑, caspase-3↑	[199]
curcumin+quercetin	K562 chronic myeloid leukemia	CDKN1B↓, AKT1↓, IFN-γ↓, BTG2↑, p21↑, p27↑, CDKN1A↑, Fas↑, FasL↑	[200]

## 5. Conclusions

Apoptosis is a protective mechanism against neoplastic transformation, as it is able to eliminate genetically damaged cells. It has been reported that its dysregulation is a biological event involved in the cancer etiopathology. In this regard, the discovery of natural compounds able to induce this death mechanism in tumor cells could be of great importance for new anticancer therapies. In recent decades, dietary polyphenols have attracted much interest due to their ability to act as both effective chemopreventive and chemotherapeutic agents. However, the anti-tumor effects observed in vitro are very often different from those obtained in vivo due to their poor bioavailability. From in vitro studies analysis, it emerged that polyphenols are able to interfere with all stages of carcinogenesis process by modulating different cellular pathways and inducing and/or inhibiting the expression of several proteins related to proliferation, migration, metastasis and apoptosis. Multifaceted actions of dietary flavonoid and non-flavonoid compounds on cell death signals modulation that involves both extrinsic and intrinsic apoptotic pathways were reported. Moreover, the polyphenols’ chemopreventive effects greatly depend on the natural compound concentration and specific tumor cell type. Interestingly, their beneficial actions increased when combined with conventional chemotherapy drugs or other dietary polyphenols. In conclusion, we believe that the increasingly detailed knowledge of the molecular apoptotic mechanisms induced by polyphenols will be very important to design new therapeutic strategies useful for cancer treatment involving polyphenols as adjuvants.

## Data Availability

Not applicable.

## Abbreviations

AGS	gastric adenocarcinoma
AKT	protein-chinasi B
APAF-1	apoptotic protease activating factor-1
ATF6	activating transcription factor 6
ATP	adenosine triphosphate
BAD	BCL-2 associated agonist of cell death
BAK	BCL-2 antagonist/killer
BAX	BCL-2-associated X protein
BCL-2	B-cell lymphoma-2
BCL-w	B-cell lymphoma-w
BCL-XL	B-cell lymphoma-extra large
BFL-1/A1	Bcl-2-related protein A1
BIC	bone-inducing protein complex
BIM	BCL-2-interacting mediator of cell death
BMF	BCL-2 modifying factor
BOC	cell adhesion associated, oncogene regulated
BTG2	BTG family member 2
c-XIAP1	cellular inhibitor of apoptosis protein 1
c-XIAP2	cellular inhibitor of apoptosis protein 2
CASPASE	cysteine-dependent aspartate-directed protease
CAT	catalase
CBX7	chromobox protein homolog 7
CDKN1A	cyclin dependent kinase inhibitor 1A
CDKN1B	cyclin-dependent kinase inhibitor 1B
c-IAP1	cellular inhibitor of apoptosis protein 1
COX2	cyclooxygenase 2
CRADD	CASP2 and RIPK1 domain containing adaptor with death domain
CYLD	ubiquitin carboxyl-terminal hydrolase
DEDD	death effector domain containing protein
DNA	deoxyribonucleic acid
DOX	doxorubicin
DR	death receptor
DR-5	death receptor 5
EGCG	epigallocatechin gallate
EGFR	epidermal growth factor receptor
ER	estrogen receptor
ERK	extracellular signal-regulated kinase
ERK1/2	extracellular signal-regulated kinase 1
ERα	estrogen receptor alpha
ERβ	estrogen receptor beta
FADD	fas-associated death domain
FAS	fas cell surface death receptor
FASL	fas ligand
FOXO3a	forkhead box O3a
GPx	glutathione peroxidase
GRP78	glucose-regulated protein 78
GSH	reduced glutathione
HNSCC	head and neck squamous cell carcinoma
HO-1	heme oxygenase-1
HONE1	epithelial tumor cell lines 1
HRK	harakiri, BCL-2 interacting protein
HTRA2	high-temperature requirement protein A2
IFN-γ	interferon gamma
IGF-IR	type 1 insulin-like growth factor receptor
JAK	janus kinase
JNK	c-Jun N-terminal kinase
LPH	phloridzine hydrolase
MAPK	mitogen-activated protein kinase
MCL-1	myeloid cell leukemia-1
MDR1	multidrug resistance protein 1
MMP	mitochondrial membrane potential
mTOR	mammalian target of rapamycin
NAC	N-acetylcysteine
ncRNAs	non-coding RNAs
NF-κB	nuclear factor kappa B
NOXA	phorbol-12-myristate-13-acetate-induced protein 1
NQO1	NAD(P)H quinone oxidoreductase
NRF2	nuclear factor erythroid 2–related factor 2
NSCLC	non-small cell lung cancer
PARP-1	poly [ADP-ribose] polymerase 1
PI3K	phoshatidylinositol-3 kinase
PKC	protein kinase C
PPARγ	peroxisome proliferator-activated receptor gamma
PTC	papillary thyroid cancer
PTEN	phosphatase and tensin homolog
PTK	protein tyrosine kinase
PUMA	p53-up-regulated modulator of apoptosis
RAGE	receptor for advanced glycation end products
ROS	reactive oxygen species
SMAC/DIABLO	second mitochondria-derived activator of caspase/direct inhibitor of apoptosis-binding protein with low isoelectric point
SOD	superoxide dismutase
STAT	signal transducer and activator of transcription
STAT3	signal transducer and activator of transcription 3
TNF	tumor necrosis factor
TNF-α	tumor necrosis factor alpha
TNF-R	tumor necrosis factor receptor
TNFRSF10A	tumor necrosis factor receptor superfamily 10A
TNFRSF10B	tumor necrosis factor receptor superfamily 10B
TNFRSF1B	tumor necrosis factor receptor superfamily member 1B
TNFRSF21	tumor necrosis factor receptor superfamily member 21
TNFRSF25	tumor necrosis factor receptor superfamily member 25
TNFSF10	tumor necrosis factor (ligand) superfamily member 10
TRADD	tumor necrosis factor receptor type 1-associated death domain protein
TRAIL	TNF-related apoptosis-inducing ligand
TRAIL-R	TNF-related apoptosis-inducing ligand receptor
XIAP	X-linked inhibitor of apoptosis
ZIP9	Zrt- and Irt-like protein 9
